# Drying Effects on Chemical Composition and Antioxidant Activity of *Lippia thymoides* Essential Oil, a Natural Source of Thymol

**DOI:** 10.3390/molecules26092621

**Published:** 2021-04-30

**Authors:** Lidiane Diniz do Nascimento, Sebastião Gomes Silva, Márcia Moraes Cascaes, Kauê Santana da Costa, Pablo Luis Baia Figueiredo, Cristiane Maria Leal Costa, Eloisa Helena de Aguiar Andrade, Lênio José Guerreiro de Faria

**Affiliations:** 1 Programa de Pós-Graduação em Engenharia de Recursos Naturais da Amazônia, Universidade Federal do Pará, Belém 66075-110, Pará, Brazil; leniojgfaria@gmail.com; 2 Coordenação de Botânica, Museu Paraense Emílio Goeldi, Belém 66077-830, Pará, Brazil; eloisa@museu-goeldi.br; 3 Instituto de Ciências Exatas e Naturais, Universidade Federal do Pará, Belém 66075-110, Pará, Brazil; profsebastiaogs@gmail.com; 4 Programa de Pós-Graduação em Química, Universidade Federal do Pará, Belém 66075-110, Pará, Brazil; cascaesmm@gmail.com; 5 Faculdade de Biotecnologia, Instituto de Biodiversidade, Universidade Federal do Oeste do Pará, Santarém 68035-110, Pará, Brazil; 6 Departamento de Ciências Naturais, Universidade do Estado do Pará, Belém 66050-540, Pará, Brazil; pablo.figueiredo@uepa.br; 7 Programa de Pós-Graduação em Engenharia Química, Universidade Federal do Pará, Belém 66075-110, Pará, Brazil; cristianemlcosta@gmail.com

**Keywords:** drying kinetics, thymol, yield, multivariate analysis, DPPH

## Abstract

Leaves of *Lippia thymoides* (Verbenaceae) were dried in an oven at 40, 50 and 60 °C and the kinetic of drying and the influence of the drying process on the chemical composition, yield, and DPPH radical scavenging activity of the obtained essential oils were evaluated. The composition of the essential oils was determined with gas chromatography-mass spectrometry and gas chromatography-flame ionization detection analyses. The influence of drying on the chemical composition of the essential oils of *L. thymoides* was evaluated by multivariate analysis, and their antioxidant activity was investigated via the 2,2-diphenyl-1-picrylhydrazyl (DPPH) assay. The Midilli model was the most appropriate to describe the behavior of drying kinetic data of *L. thymoides* leaves. Thymol was the major compound for all analyzed conditions; the maximum content was obtained from fresh leaves (62.78 ± 0.63%). The essential oils showed DPPH radical scavenging activity with an average of 73.10 ± 12.08%, and the fresh leaves showed higher inhibition (89.97 ± 0.31%). This is the first study to evaluate the influence of drying on the chemical composition and antioxidant activity of *L. thymoides* essential oils rich in thymol.

## 1. Introduction

Essential oils and their constituents have been described as antioxidants [1,2,3,4,5], food preservatives [6,7,8], and mosquito repellents [9,10,11]. Essential oils containing thymol as a major component have been used in the industry as flavorings and food preservatives, and even in aromatherapy [4,12,13]. This compound has been registered by the European Commission for use as flavoring in foodstuffs; it is also used as a food additive in the USA and China [12,14]. Furthermore, isolated thymol as well as essential oils with high levels of thymol demonstrated a protective action in the treatment of soybean seeds [15]; they also exhibit antifungal [16,17,18], antibacterial [19,20], antiviral [21], and antioxidant activities [13]. A previous study investigated the antioxidant activity of some pure essential oil components using lipid model systems and concluded that phenolic compounds were efficient antioxidants and that thymol was one of the most active components in the class of oxygenated monoterpenes [22]. Furthermore, thymol has been indicated as an alternative to synthetic antioxidants in the food matrix [14].

*Lippia thymoides* Mart. & Schauer (syn *Lippia micromera* var. *tonsilis* Moldenke) belongs to Verbenaceae [23,24,25] and has been extensively investigated for the biological activities associated with its oil or extract. Silva et al. (2015) [26] validated the traditional use of *L. thymoides* for the treatment of wounds and fever. The dichloromethane fraction showed antimicrobial activities against the species *Bacillus cereus*, *Candida parapsilosis*, and *Staphylococcus aureus* [26]. In addition, Pinto et al. (2013) [27] evaluated the methanolic extracts of leaves, branches, and flowers of *L. thymoides*, which exhibited antimicrobial activity against *Staphylococcus aureus*; these results corroborated its traditional use in the treatment of infectious diseases [27]. Despite the importance of this species in traditional medicine, few studies have reported the chemical composition of *L. thymoides* essential oil [28,29,30,31].

The drying process prolongs the shelf life of products, inhibiting chemical and enzymatic reactions. However, drying can cause changes in the yield of essential oils, polyphenols, pigments, and vitamin contents as well as appearance, taste, and color [32]. Different drying techniques have been applied to various aromatic plants to evaluate the effect of drying on the color, composition, and yield of the essential oils as well as to determine the best method for drying a specific plant [33,34,35,36,37].

The drying process can directly affect the composition and yield of essential oils, but this varies according to each species and drying method applied. In the present study, we evaluated for the first time the effect of the air temperature on the thin-layer drying kinetics and the influence of different drying conditions on the chemical composition, yield, and antioxidant activity of the essential oil of *Lippia thymoides* Mart. & Schauer (Verbanaceae) rich in thymol.

## 2. Results and Discussion

### 2.1. Drying Kinetics

**Figure 1:**
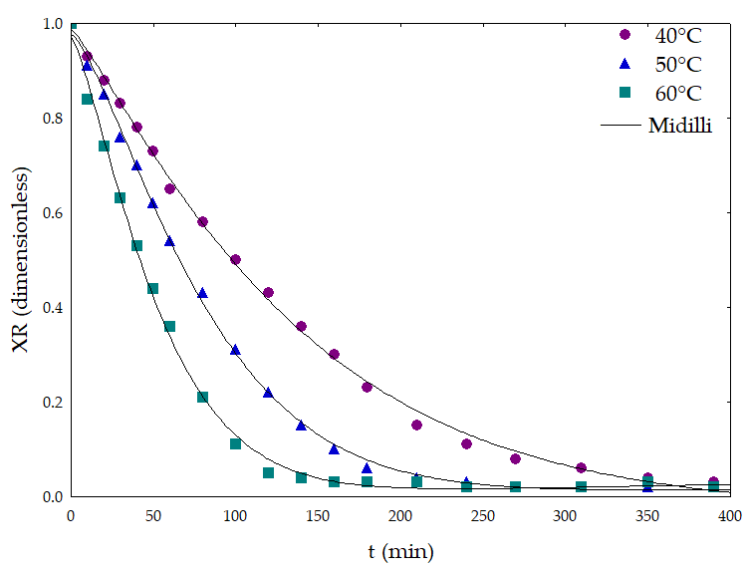
Values of moisture ratio versus time in thin-layer drying of *Lippia thymoides* leaves, adjusted using the Midilli model.

The initial moisture content of *L. thymoides* fresh leaves was 68.15 ± 0.01% on a wet basis. At the end of the drying process, the moisture content was 31.28 ± 1.51% for the 40 °C conditions, 12.34 ± 0.09% at 50 °C, and 9.29 ± 0.07% at 60 °C. During the drying of *L. thymoides* leaves, the moisture ratio decreased exponentially, and an increase in the air temperature led to higher water removal from plant matrices (Figure 1). To explain a similar behavior, Perea-Flores et al. (2012) [38] reported that during the falling period, the surface of the material was no longer saturated with water, and that the phenomenon of internal diffusion controlled the drying rate [38]. The kinetic curves in Figure 1 presented a typical pattern of drying plots [39,40,41]. In the present study, the moisture ratio reached stable values in the range of 0.03 after 350 min (40 °C), 210 min (50 °C), and 160 min (60 °C), as presented in Figure 1. In this condition, we obtained a moisture content of 6.54% on a wet basis. The pattern of the kinetic drying curves described in Figure 1 is in accordance with the results previously described for *Ocimum basilicum* [42,43] and *Warionia saharae* [44].

Kucuk et al. (2014) [45] reported that different models have been applied to predict drying conditions of several products [45]. The Midilli model presented the best fit of the experimental drying data at different temperatures (Table 1, Figure 1), and presented *SEE* < 0.02% and *MRE* < 1.18%. The *R^2^* values were 99.91% (40 °C), 99.95% (50 °C), and 99.87% (60 °C). The Midilli model is indicated to describe the thin layer drying process [45] and it was appropriated to describe the drying kinetic of *L. thymoides*. Midilli was also suitable for describing the drying curves of leaves of *Piper umbellatum* [46], *Ocimum basilicum* [43], and *Vernonia amygdalina* [47].

According to Table 1, for the Lewis, Diffusion approach, and Henderson & Pabis models, the “*k*” values increased as the drying temperature increased. However, for the Page and Midilli models, the temperature variation (40, 50 and 60 °C) did not cause a defined trend in the values of “*k*”. The parameter “*k*” is defined as the constant of drying (min^−1^) and according to Dorneles et al. (2019) [46], it is related to the effective diffusivity during the falling period of kinetic drying, in which liquid diffusion controls the process [46].

### 2.2. Drying Effects on Essential Oil Yield

The drying conditions had a significant effect on the yield of the essential oils (*p* < 0.05) (Table 2). The yield obtained from fresh leaves of *L. thymoides* was similar to the yield obtained by drying for 390 min at 60 °C (0.50% on average). The highest yields were obtained for dried leaves at 40 °C and 50 °C (0.74% on average). Therefore, according to the experimental conditions here described, to achieve better yields, it is necessary to reduce the moisture content of *L*. *thymoides* leaves, but the samples should be dried at temperatures in the range of 40 to 50 °C. At higher temperatures such as 60 °C, there is a greater loss in the components by volatilization, resulting in lower yields during extraction.

Considering the storage structures found in different organs and the volatile constituents of the aromatic plants, the selection of the appropriate drying methods is important because drying can affect the yield and the chemical composition of the essential oil [35,48]. We did not find previous studies evaluating the effect of different drying conditions on the yield of essential oil of *L. thymoides*, however, Ebadi et al. (2015) [49] investigated the drying of *Lippia citriodora* leaves using different methodologies including oven-drying (40, 50, and 60 °C), and they found that the increase in temperature generated damage to glandular trichomes, which may have favored the loss of volatiles and the reduction in the yield [49]. Mashkani et al. (2018) [50] subjected the leaves of *Thymus daenensis* to different drying methods (sun and shade, oven, microwave, and vacuum drying) and observed that, in general, there was a reduction in the yield of essential oils at increased temperatures [50]. Alves et al. (2015) [51] investigated the influence of *Ocimum basilicum* leaf-drying on the yield and composition of the essential oil and observed that the oil yield decreased linearly over eight days at 40 °C (6.0% to 3.9%) [51]. Therefore, an increase in the temperature contributes to high evaporation rates, which can damage the cells that store the essential oil, intensifying the release of volatile compounds and resulting in lower yields after extraction.

### 2.3. Drying Effects on Chemical Composition

Thirty-two volatile constituents were identified, comprising a range of 90.07 ± 3.11% to 98.53 ± 0.05% of the total composition of the analyzed essential oils (Table 2). Thymol was the major compound obtained from both fresh and dried leaves. The highest content was observed in fresh leaves (62.78 ± 0.63%), while the levels in the extracted essential oils after drying at 40, 50 and 60 °C were 49.21 ± 11.46%, 53.03 ± 11.76%, and 59.29 ± 2.89%, respectively.

Besides thymol, the main components identified in the *L. thymoides* essential oil were *p*-cymene (2.97 ± 2.79%–8.97 ± 0.64%), γ-terpinene (2.75 ± 3.55%–12.36 ± 4.64%), thymyl acetate (4.92 ± 0.10%–7.22 ± 2.30%), and *E*-caryophyllene (5.21 ± 0.44%–8.84 ± 1.10%). The oxygenated monoterpenes were the predominant chemical class, reaching the highest level in fresh leaves (72.95 ± 2.89%). These results were in agreement with previously published data on the composition of *L. thymoides* essential oils [30,31], but differed from the composition described by Silva et al. (2016) [29], who found an essential oil with high levels of β-caryophyllene (17.22–26.27%), borneol (4.45–7.36%), camphor (3.22–8.61%), camphene (2.64–5.66%), and germacrene D (4.72–6.18%) [29].

**Figure 2:**
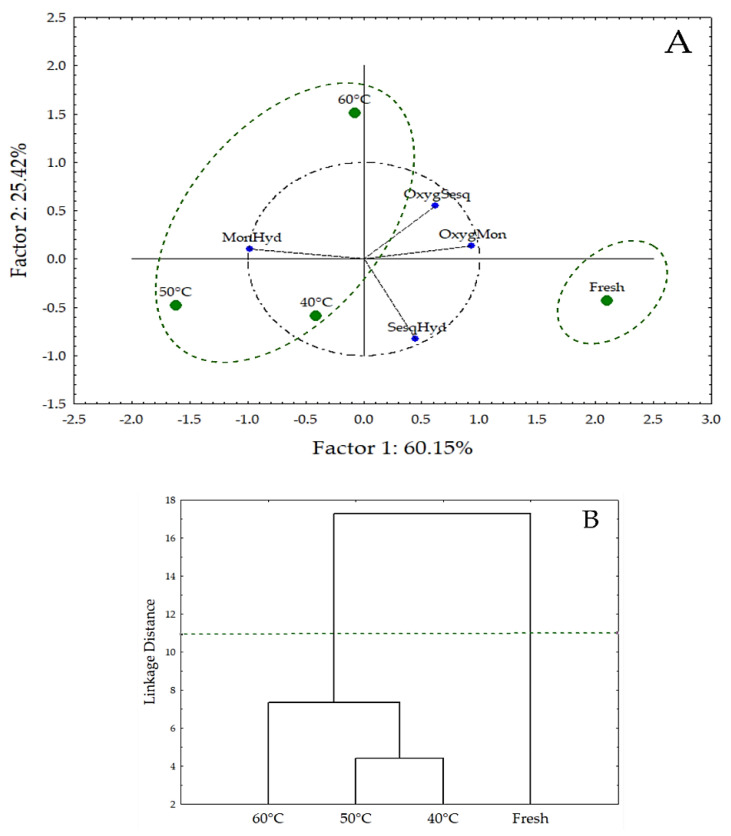
Multivariate analysis using data from the chemical composition of essential oils obtained from fresh and dried *Lippia thymoides* leaves. (**A**) Principal component analysis (PCA); MonHyd: Monoterpene hydrocarbons; OxygSesq: Oxygenated sesquiterpene; OxygMon: Oxygenated monoterpenes; SesqHyd: Sesquiterpene hydrocarbons. (**B**) Hierarchical cluster analysis (HCA). The dashed green lines indicate the identified groupings.

Here, we utilized the multivariate analysis to investigate possible similarities between the chemical classes of the constituents identified in the essential oils of the fresh and dried leaves of *L. thymoides* (Table 2). From the PCA data (Figure 2A), we deducted a fitting of 85.57% (PC1 + PC2) of the chemical variation in the samples. The principal components (PC1 and PC2) divided the chemical composition of the fresh and dried leaves into two groups (Figure 2A).

These results confirmed that regardless of the applied drying temperature (40, 50 and 60 °C), the essential oils extracted from the dried leaves had a similar chemical profile and formed Group 1. The percentages of monoterpene hydrocarbons increased in essential oils extracted from leaves after the drying process when compared to fresh leaves. However, for the class of oxygenated monoterpenes, a reduction in their contents was detected when compared to oil extracted from fresh leaves (control). This variation may have favored the formation of Group 2, composed only by the chemical constituents of the fresh leaves, which was confirmed by the analysis of the dendrogram in Figure 2B.

Previously, a drying study described the effect of temperature and air velocity of drying on the composition of the *Piper umbellatum* leaves. They also reported a significant difference between the chemical profile of the fresh and dried leaves, but they found little difference between the samples dried at 40, 50, 60 and 70 °C (0.4 and 0.7 m.s^−1^) [46].

Other studies have also evaluated the effect of drying on monoterpene content and reported an effect similar to that described in the present study. Mashkani et al. (2018) [50] evaluated different drying methods for *Thymus daenensis* and observed that when compared with fresh plants, oven-drying at 35 °C after a pre-drying operation resulted in a higher content in monoterpene hydrocarbons and reduced the oxygenated monoterpenes (especially the content of thymol and carvacrol) [50]. Shahhoseini et al. (2013) [54] subjected the aerial flowering parts of *Lippia citriodora* to different drying methods and observed that during oven-drying, the percentage of monoterpene hydrocarbons increased and those of oxygenated monoterpenes decreased with an increase in the drying temperature [54].

The thymol content decreased after the drying process when compared to the content presented in the fresh leaves. Similarly, Calín-Sánchez et al. (2013) [55] evaluated the effects of different methodologies and temperatures in the chemical composition of *Thymus vulgaris* essential oils, which presented high levels of thymol. They found 732 mg·100g^−1^ of thymol in the fresh leaves and after oven-drying, the levels changed to 571 mg·100g^−1^ (40 °C), 479 mg·100g^−1^ (50 °C), and 451 mg·100g^−1^ (60 °C) [55]. In the study with the essential oil of *Lippia origanoides*, Queiroz et al. (2018) [56] also observed a reduction in thymol content after the drying process [56]. Therefore, the authors found that the drying process promoted a reduction in the levels of thymol, which corroborate with our results.

### 2.4. Antioxidant Activity

The DPPH radical scavenging activity of the essential oils of *L. thymoides* obtained from fresh and dried leaves (40 °C, 50 °C, and 60 °C) are listed in Table 3. The variation in drying temperature did not show a defined trend in the results of antioxidant activity, but the highest percentage of inhibition of the DPPH radical was observed for the essential oil sample from the fresh material (89.97 ± 0.31%), which presented the highest percentage of thymol (62.78%, Table 2). In our study, we observed that the samples of the essential oils obtained after the drying process showed lower antioxidant activity, which may be associated with lower levels of thymol. A previous study investigated the antioxidant activity of some pure essential oil components using lipid model systems, and the authors concluded that the phenolic compounds were efficient antioxidants and they considered thymol as one of the most active components in the class of oxygenated monoterpenes [22]. It is interesting to note that thymol has been indicated as an alternative to synthetic antioxidants in the food matrix [14].

Although the chemical composition of the essential oils extracted after drying presented high similarity (Figure 2B), only the oils of *L. thymoides* extracted from dried leaves at 40 °C and 60 °C inhibited the DPPH radical in a statistically similar manner (Table 3). Natural products such as extracts and essential oils are formed by a complex mixture of organic compounds that act synergistically, increasing the biological activity or even antagonistically, thus reducing the verified activity, so identifying the compounds involved with a given biological response represents a methodological challenge [57,58,59]. Based on this, we conjecture that this variation in the results of DPPH inhibition percentage for the dried leaves could be involved with the synergist or antagonist effects (Table 3). Other studies identified synergist effects in the antioxidant [60] and antibacterial [61,62,63] activities of essential oils. For example, Ranjbaran et al. (2019) [13] reported that the high antioxidant activity of *Trachyspermum ammia* essential oil could be associated with the strong synergism between the monoterpenes (*p*-cymene and *γ*-terpinene), the phenolic monoterpenes (thymol), and the oxygenated monoterpenes [13].

Essential oils with high thymol contents have been evaluated for their DPPH radical scavenging activities such as the essential oil from *Trachyspermum ammia* [13]. This essential oil showed robust antioxidant activity, which may be associated with its high content of thymol (40.25%). Using the DPPH method, the IC_50_ were 54 ± 3.2 µg·mL^−1^ and 90 ± 5.4 µg·mL^−1^ for the essential oil and thymol, respectively [13]. The essential oil of oregano (*Origanum vulgare*) with thymol (45%) and carvacrol (37.4%) as the major compounds was subjected to DPPH radical scavenging tests and resulted in IC_50_ values between 0.76 µg·mL^−1^ and 0.33 µg·mL^−1^, indicating that this essential oil is an interesting natural source of antioxidant compounds with applications in the chemical, pharmaceutical, cosmetics, and food industries [64].

Few studies have reported the antioxidant profile of the essential oil of the species *L. thymoides*. Silva et al. (2016) [29] applied scavenging of the DPPH free radical and β-carotene bleaching tests to determine the antioxidant potential of the *L. thymoides* essential oil, rich in *E*-caryophyllene (17.22–26.27%). The authors did not find a satisfactory antioxidant activity for the essential oil, with EC_50_ (concentration that caused 50% of the DPPH radical scavenging) values more than 236 mg·mL^−1^ in the DPPH assay and a reduction of less than 11% in the β-carotene bleaching test [29]. There is a growing interest in the development of antioxidant substances, mainly from natural products [65].

## 3. Material and Methods

### 3.1. Plant Material

Leaves of *L. thymoides* were collected in the municipality of Abaetetuba (State of Pará, Amazon region, Brazil). The botanical identification (MG 213373) was performed in the Herbarium of the Museu Paraense Emílio Goeldi (Belém, State of Pará, Brazil).

### 3.2. Thin-Layer Drying Kinetics of Lippia thymoides Leaves

The drying kinetics were measured at 40, 50 and 60 °C using 12.03 ± 0.05 g of fresh leaves. All assays were conducted in duplicate for 390 min using a convection oven (model SL-102, Solab, Piracicaba, São Paulo, Brazil) until reaching the equilibrium moisture content. The moisture content was determined according to the standard methods of analysis [66] at 105 °C. After the experiments, the moisture ratio (*XR*) was calculated according to previous studies [42,46,67].

Some mathematical models applied to describe the experimental drying kinetics of natural products were selected (Table 4): Lewis, Henderson & Pabis, Page, Diffusion approach, Midilli, and Wang & Singh, using a nonlinear regression procedure to estimate the parameters associated with each model.

### 3.3. Drying Conditions Versus Yield and Chemical Composition

Samples of 90.11 ± 0.06 g of fresh leaves were dried at 40 °C, 50 °C, and 60 °C for 390 min in a convection oven. Then, the dried samples were subjected to hydrodistillation.

#### 3.3.1. Essential Oil Extraction

Leaves of *L. thymoides* (fresh and dried) were subjected to hydrodistillation using a modified Clevenger glass extractor for 3 h, as described by Nascimento et al. (2020) [73]. We utilized 30 g of plant material for each experiment. The yield (%) of the essential oil was expressed as the percentage of oil in relation to the dry matter of the leaves.

#### 3.3.2. Analysis of Chemical Composition

The chemical composition was analyzed with gas-phase chromatography coupled to mass spectrometry (GC/MS) using a Shimadzu QP 2010 Plus System (Shimadzu Corporation, Kyoto, Japan), equipped with silica capillary columns (DB-5MS, length of 30 m, inner diameter of 0.25 mm, and film thickness of 0.25 μm); carrier gas: helium, linear velocity: 36.5 cm·s^−1^; type of injection: splitless (solution of 2 μL of oil in 500 μL of hexane); injector temperature: 250 °C, temperature range: 60 °C to 250 °C, gradient of 3 °C·min^−1^; electron impact mass spectrometry: 70 eV; ion source temperature and connection parts: 220 °C. The quantification of each component was performed by peak-area normalization using a flame ionization detector (GC-FID, QP 2010 system, Shimadzu Corporation, Kyoto, Japan) under the same conditions as GC/MS, except for the carrier gas, which was hydrogen.

The components were identified based on the retention index (RI), which was calculated using the retention times of a homologous series of n-alkanes (C8-C40, Sigma-Aldrich, San Luis, Missouri, USA). The pattern of fragmentation observed in the spectra was compared with existing patterns of authentic samples in data system libraries and the literature [52].

#### 3.3.3. 2,2-Diphenyl-1-picrylhydrazyl (DPPH) Free Radical Scavenging Assay

The antioxidant activity of the essential oils was evaluated using the 2,2-diphenyl-1-picrylhydrazyl (DPPH) radical scavenging method. A stock solution of DPPH (0.5 mM) was prepared in ethanol. The solution was diluted to approximately 60 µM, measuring an initial absorbance of 0.62 ± 0.02 at 517 nm at 22 °C. The absorbance was measured at the start of the reaction, every 5 min during the first 30 min, and then at 30 min intervals until a constant absorbance value (plateau of reaction, 2 h). Each essential oil sample (50.0 µL, 20 mg·mL^−1^) was mixed with a Tween 20 solution (0.5%, 50 µL, *w/w*), and added to DPPH (0.5 mM, 1900 µL) in ethanol. The control sample was prepared in the same conditions, replacing the essential oil sample by ethanol. The standard curves were prepared using Trolox (6-hydroxy-2,5,7,8-tetramethylchroman-2-carboxylic acid) (Sigma-Aldrich, San Luis, Missouri, USA) at concentrations of 30 µg·mL^−1^, 60 µg·mL^−1^, 150 µg·mL^−1^, 200 µg·mL^−1^, and 250 µg·mL^−1^. The DPPH inhibition percentage and total antioxidant activity were expressed as milligrams of Trolox (mgTE·g^−1^), as described by Choi et al. (2000) [74] and Figueiredo et al. (2019) [75].

### 3.4. Statistical Analyses

The most appropriate models to describe the drying kinetics curves were chosen based on the mean relative error (MRE, Equation (1)), standard error of estimate (SEE, Equation (2)), and coefficient of determination (R^2^). A MRE value of less than 10% was one of the criteria for selecting the best model [76]. Statistical analyses were conducted using Statistica^®^ 13.1 (TIBCO Software Inc., Palo Alto, CA, USA), and the Quasi-Newton method was applied. N represents the number of experiments, df the degrees of freedom, XR_exp_ the experimental moisture ratio, and XR_pre_ the predicted moisture ratio.

(1) $$\mathrm{MRE}=\frac{100}{N}\;\overset{N}{\underset{i=1}{\sum }}\left|\frac{{\mathrm{XR}}_{\exp}-{\mathrm{XR}}_{\mathrm{pre}}}{{\mathrm{XR}}_{\exp}}\right|$$

(2) $$\mathrm{SEE}=\;\sqrt{\frac{\sum_{i=1}^{N}{\left({\mathrm{XR}}_{\exp}-{\mathrm{XR}}_{\mathrm{pre}}\right)}^{2}}{\mathrm{df}}}$$

The yield and antioxidant activity data were subjected to variance analysis (ANOVA) and their means were compared performing the Tukey test (at a significance level of *p* < 0.05). Principal component analysis (PCA) was performed using a matrix correlation. In addition, the Euclidean distance and complete binding were used for the hierarchical cluster analysis (HCA) of the samples. The statistical analyses were conducted using Statistica^®^ 13.1 (TIBCO Software Inc., Palo Alto, CA, USA).

## 4. Conclusions

The study of drying kinetics provides data for the selection of the type of dryer as well as the best operating drying conditions. Midilli was the best model to describe the drying kinetics of *L. thymoides* leaves. In the present study, the drying conditions reached an appropriate moisture content for safe storage. The highest yields of essential oil were obtained from leaves that were dried at 40 °C and 50 °C after drying for 390 min. The chemical composition showed a similar quantitative profile for all the drying conditions (40, 50 and 60 °C), but it differed from the chemical composition of the fresh material.

Thymol was the major compound identified in the essential oil obtained from fresh and dried leaves. The highest thymol content (62.78 ± 0.63%) and inhibition percentage of the DPPH radical (89.97 ± 0.31%) were obtained from fresh leaves. Thus, *L. thymoides* can be a natural source of thymol, with levels greater than 50% of the composition of the essential oil.

## Figures and Tables

**Table 1:** Parameters of the models applied to the experimental data analyses of the drying kinetic curves of *Lippia thymoides* leaves.

Models	Temperature (°C)	Parameters				*R*^2^ (%)	*MRE* (%)	*SEE* (%)
		*k*						
Lewis	40	0.0076				99.504	17.0104	0.0333
	50	0.0115				99.274	27.8596	0.0423
	60	0.0179				99.492	0.8904	0.0329
		*a*		*k*				
Henderson & Pabis	40	1.039		0.0079		99.627	14.0965	0.0298
	50	1.0611		0.0122		99.485	19.4258	0.0367
	60	1.0351		0.0185		99.552	3.1528	0.0318
		*n*		*k*				
Page	40	1.1787		0.0032		99.833	2.5868	0.0159
	50	1.2794		0.0033		99.923	12.0830	0.0141
	60	1.2076		0.0077		99.780	25.8821	0.0214
		*a*	*b*	*k*				
Diffusion approach	40	−0.6480	0.5837	0.0173		99.890	5.0964	0.0161
	50	−2.0200	0.7479	0.0248		99.926	8.3433	0.0143
	60	−2.9001	0.8332	0.0337		99.815	24.5185	0.0211
		*a*	*b*	*n*	*k*			
Midilli et al.	40	0.9880	−5.5 × 10^−5^	1.1638	0.0032	99.912	1.1798	0.0157
	50	0.9777	−3.8 × 10^−5^	1.3538	0.0023	99.951	0.9000	0.0120
	60	0.9721	−6.6 × 10^−5^	1.2969	0.0053	99.877	0.9241	0.0177
		*a*		*b*				
Wang & Singh	40	−0.0058		9 × 10^−6^		99.920	1.1627	0.0141
	50	−0.0077		1.4 × 10^−5^		99.017	25.2498	0.0506
	60	−0.0092		1.9 × 10^−5^		92.270	62.1788	0.1297

Note: *k* is the drying constant (min^−1^); *a*, *b*, and *n* are the model coefficients; *MRE:* mean relative error; *SEE:* standard error of estimate; and *R^2^*: coefficient of determination.

**Table 2:** Yield, chemical constituents and their percentage variation in the essential oils of *Lippia thymoides* leaves after the drying process at 40, 50, and 60 °C.

			Fresh	40 °C	50 °C	60 °C
		Yield * (%)	0.53 ^a^ ± 0.01	0.72 ^b^ ± 0.01	0.76 ^b^ ± 0.00	0.46 ^a^ ± 0.09
RI_L_	RI_C_	Compound	Area %	Area %	Area %	Area %
924	927	α-thujene	-	0.78 ± 0.04	0.27 ± 0.38	0.62 ± 0.23
988	985	Myrcene	0.52 ± 0.62	2.46 ± 1.39	2.10 ± 1.15	2.16 ± 0.48
1002	1005	α-phellandrene	-	0.26 ± 0.00	0.11 ± 0.15	0.23 ± 0.02
1014	1012	α-terpinene	0.37 ± 0.45	1.70 ± 0.97	1.39 ± 0.76	1.40 ± 0.13
1020	1019	*p*-cymene	2.97 ± 2.79	8.75 ± 3.08	8.36 ± 3.54	8.97 ± 0.64
1022	1025	*o*-cymene	0.04 ± 0.05	0.35 ± 0.49	2.28 ± 0.57	-
1044	1042	*E*-β-ocimene	-	0.04 ± 0.05	0.08 ± 0.11	0.12 ± 0.01
1054	1056	γ-terpinene	2.75 ± 3.55	9.54 ± 0.59	12.36 ± 4.64	8.19 ± 0.28
1065	1064	cis-sabinene hydrate	0.05 ± 0.06	0.02 ± 0.03	-	0.18 ± 0.00
1086	1088	Terpinolene	-	0.08 ± 0.12	0.20 ± 0.04	0.17 ± 0.01
1095	1099	Linalool	0.03 ± 0.04	0.13 ± 0.18	0.27 ± 0.07	0.19 ± 0.00
1098	1104	trans-sabynene hydrate	-	0.05 ± 0.07	0.04 ± 0.05	-
1141	1146	Camphor	-	0.02 ± 0.00	0.03 ± 0.00	-
1167	1175	Umbellulone	0.88 ± 0.04	1.48 ± 2.02	0.06 ± 0.02	0.28 ± 0.36
1174	1180	Terpinen-4-ol	0.73 ± 0.13	1.05 ± 1.01	1.44 ± 0.10	-
1232	1237	Thymol methyl ether	1.28 ± 0.30	2.69 ± 1.54	1.47 ± 0.09	1.28 ± 0.03
1289	1297	Thymol	62.78 ± 0.63	49.21 ± 11.46	53.03 ± 11.76	59.29 ± 2.89
1349	1356	Thymyl acetate	7.22 ± 2.30	6.46 ± 0.72	5.25 ± 0.45	4.92 ± 0.10
1374	1379	α-copaene	-	0.02 ± 0.02	0.04 ± 0.01	0.07 ± 0.00
1387	1388	β-bourbonene	-	0.02 ± 0.00	0.02 ± 0.02	0.03 ± 0.00
1417	1425	*E*-caryophyllene	8.84 ± 1.10	8.08 ± 0.26	7.46 ± 0.56	5.21 ± 0.44
1432	1439	α-trans-bergamotene	0.23 ± 0.04	0.39 ± 0.34	0.13 ± 0.01	-
1452	1458	α-humulene	1.49 ± 0.22	1.75 ± 1.00	0.89 ± 0.09	0.79 ± 0.07
1484	1486	Germacrene D	0.59 ± 0.13	0.79 ± 0.26	0.37 ± 0.07	0.40 ± 0.02
1495	1499	γ-amorphene	-	0.05 ± 0.06	0.09 ± 0.00	0.24 ± 0.00
1500	1504	α-muurolene	-	0.20 ± 0.17	0.07 ± 0.01	0.14 ± 0.01
1513	1519	γ-cadinene	0.42 ± 0.14	0.36 ± 0.28	0.15 ± 0.02	0.22 ± 0.01
1522	1528	δ-cadinene	0.78 ± 0.24	0.77 ± 0.63	0.30 ± 0.03	0.41 ± 0.04
1582	1589	Caryophillene oxide	1.09 ± 0.40	1.01 ± 0.86	0.30 ± 0.07	1.06 ± 0.05
1608	1615	Humulene epoxide II	-	0.02 ± 0.03	0.01 ± 0.01	0.14 ± 0.01
1640	1646	epi-α-murrolol	-	0.02 ± 0.02	-	0.07 ± 0.02
1652	1659	α-cadinol	-	0.02 ± 0.03	-	0.07 ± 0.01
		Monoterpene hydrocarbons	6.70 ± 7.49	23.95 ± 4.35	27.14 ± 11.35	21.85 ± 1.80
		Oxygenated monoterpenes	72.95 ± 2.89	61.1 ± 7.50	61.56 ± 11.08	66.14 ± 1.38
		Sesquiterpene hydrocarbons	12.35 ± 1.91	12.41 ± 2.32	9.49 ± 0.85	7.5 ± 0.59
		Oxygenated sesquiterpene	1.1 ± 0.42	1.07 ± 0.78	0.31 ± 0.06	1.33 ± 0.09
		Total **	90.07 ± 3.11	98.53 ± 0.05	98.49 ± 1.17	96.79 ± 0.26

Notes: * Columns with the same letter did not differ by the Tukey test at 5% probability; RI_(L)_: Retention index from literature [52,53]; RI_(c)_: Retention index calculated using an *n*-alkane standard solutions (C8–C40) in column DB5-MS; ** Relative percentage areas calculated based on the peak areas.

**Table 3:** Antioxidant activity of the essential oils of the fresh and dried leaves of *Lippia thymoides*.

	Antioxidant Activity *	
Sample	Trolox Equivalent (mgTE·mL^−1^)	Inhibition (%)
Fresh	231.26 ^a^ ± 0.79	89.97 ^a^ ± 0.31
40 °C	163.31 ^b^ ± 12.96	63.53 ^b^ ± 5.04
50 °C	189.26 ^c^ ± 5.38	73.63 ^c^ ± 2.09
60 °C	167.80 ^b^ ± 7.02	65.28 ^b^ ± 2.74

* Values are expressed as mean ± standard deviation (*n* = 3). Values followed by the same letter did not differ by the Tukey test at 5% probability.

**Table 4:** Mathematical models adjusted to the drying curves of *Lippia thymoides* leaves.

Models	Equations	References
Lewis	XR = exp(−k × t)	[68]
Henderson & Pabis	XR = a × exp(−k × t)	[68,69]
Page	XR = exp(−k × t^n^)	[70]
Diffusion approach	XR = a × exp(−k × t) + (1 − a) × exp(−k × b × t)	[38,71]
Midilli	XR = a × exp(−k × t^n^) + b × t	[72]
Wang & Singh	XR = 1 + a × t + b × t^2^	[69,71]

Note: *k* is the drying constant (min^−1^); a, b, and n are the model coefficients.

## Data Availability

Data is contained within the article.
